# Myeloid Targeted Human *MLL-ENL* and *MLL-AF9* Induces cdk9 and bcl2 Expression in Zebrafish Embryos

**DOI:** 10.1371/journal.pgen.1011308

**Published:** 2024-06-03

**Authors:** Alex J. Belt, Steven Grant, Robert M. Tombes, Sarah C. Rothschild

**Affiliations:** 1 Life Sciences, Virginia Commonwealth University, Richmond, Virginia, United States of America; 2 Massey Cancer Center, Virginia Commonwealth University, Richmond, Virginia, United States of America; 3 Biology, Virginia Commonwealth University, Richmond, Virginia, United States of America; VCU Massey Cancer Center, UNITED STATES

## Abstract

Acute myeloid leukemia (AML) accounts for greater than twenty thousand new cases of leukemia annually in the United States. The average five-year survival rate is approximately 30%, pointing to the need for developing novel model systems for drug discovery. In particular, patients with chromosomal rearrangements in the mixed lineage leukemia (MLL) gene have higher relapse rates with poor outcomes. In this study we investigated the expression of human MLL-ENL and MLL-AF9 in the myeloid lineage of zebrafish embryos. We observed an expansion of *MLL* positive cells and determined these cells colocalized with the myeloid markers *spi1b*, *mpx*, and *mpeg*. In addition, expression of *MLL-ENL* and *MLL-AF9* induced the expression of endogenous *bcl2* and *cdk9*, genes that are often dysregulated in MLL-r-AML. Co-treatment of *lyz*: *MLL-ENL* or *lyz*:*MLL-AF9* expressing embryos with the BCL2 inhibitor, Venetoclax, and the CDK9 inhibitor, Flavopiridol, significantly reduced the number of *MLL* positive cells compared to embryos treated with vehicle or either drug alone. In addition, cotreatment with Venetoclax and Flavopiridol significantly reduced the expression of endogenous *mcl1a* compared to vehicle, consistent with AML. This new model of MLL-r-AML provides a novel tool to understand the molecular mechanisms underlying disease progression and a platform for drug discovery.

## Author summary

Acute myeloid leukemia (AML) accounts for over twenty thousand new cases of leukemia per year in the United States. MLL rearrangements (MLL-r) occur in approximately 10% of AML patients, with 34% of those harboring an MLL-ENL or MLL-AF9 rearrangement. Patients with an MLL-r have a higher relapse rate and their overall survival is low, pointing to a need for novel therapeutic interventions. Zebrafish have emerged as a powerful model system to study hematological malignancies due to their conserved hematopoietic program, genetic tractability, and pharmacological accessibility. Expression of human *MLL-ENL* and *MLL-AF9* in the myeloid lineage of zebrafish embryos induced an expansion of myeloid cells and caused an increase in expression of endogenous *bcl2* and *cdk9*. *MLL* expression was significantly reduced in *lyz*:*MLL-ENL* and *lyz-MLL-AF9* embryos co-treated with the BCL2 inhibitor (Venetoclax) and the CDK9 inhibitor (Flavopiridol) compared to embryos treated with vehicle or either drug alone. Reduced *MLL* expression occurred in conjunction with a significant decrease in *mcl1a* expression, consistent with human AML cells. Taken together, these results further support the use of zebrafish to model hematological malignancies for drug discovery.

## Introduction

Acute myeloid leukemia (AML) is characterized by an accumulation of hematopoietic precursor cells in the bone marrow and blood as a result of multiple gene mutations and acquisition of chromosomal rearrangements [1]. In AML, mixed lineage leukemia (MLL1 or KMT2A) frequently undergoes chromosomal rearrangements resulting in the production of an oncogenic fusion protein comprised of the N-terminus of MLL and the C-terminal portion of a fusion partner [2,3]. Although many MLL rearrangements (MLL-r) have been identified, approximately half of MLL-r-AML patients harbor a genetic fusion with AF4, AF9, AF10, or ENL [3,4]. These proteins are important components of the AF4 super elongation complex (SEC) which also includes the positive transcription elongation factor b (p-TEFb) complex, bromodomain containing 4 (BRD4), and DOT1 like histone lysine methyltransferase (DOT1L). Ectopic expression of these fusion proteins leads to aberrant histone 3 lysine 79 (H3K79) methylation via DOT1L, as well as increased transcriptional elongation through p-TEFb. [5]. Patients harboring an MLL-r have a higher relapse rate and their overall survival is low [3]. Therefore, new therapeutic treatment protocols need to be investigated for patients with MLL-r AML.

Zebrafish have emerged as a powerful model system to study the etiology of hematological malignancies due to their conserved regulation of normal and malignant hematopoiesis, genetic tractability, and established imaging and pharmacologic techniques [6–13]. Expression of known fusion proteins in the myeloid lineage induces the expansion and clustering of myeloid cells in embryos and in cases where embryos survive, malignancy in adults [10,14–25]. Pharmacological inhibitors are effective in preventing the myeloid proliferation and expansion observed in embryos ectopically expressing human fusion proteins [10,23,26]. Therefore, zebrafish embryos are a valuable model system in the fight against AML.

In this study, expression of human *MLL-ENL* and *MLL-AF9* in the myeloid lineage resulted in expansion and clustering of *MLL* positive cells on the yolk of zebrafish embryos. Marker analysis confirmed colocalization of *MLL* with myeloid markers *sp1b*, *mpx*, and *mpeg* but not *lyz*. Further examination identified increased expression of endogenous *cdk9* and *bcl2* on the yolk of *MLL-ENL* and *MLL-AF9* expressing embryos but not in controls. Co-incubation of *MLL-ENL* or *MLL-AF9* expressing embryos with Venetoclax, a BCL2 inhibitor, and Flavopiridol, a CDK9 inhibitor, resulted in significantly fewer *MLL-ENL* positive cells than treatment with vehicle or either drug alone. Therefore, this study demonstrates that expression of *MLL-ENL* or *MLL-AF9* in the myeloid lineage induced an expansion of myeloid lineage cells as well as endogenous *cdk9* and *bcl2* expression in zebrafish embryos, and that cooperative inhibition of Bcl2 and Cdk9 is an effective treatment for MLL-r myeloproliferation.

## Results

### Myeloid expression of MLL-ENL in zebrafish embryos

**Fig 1:**
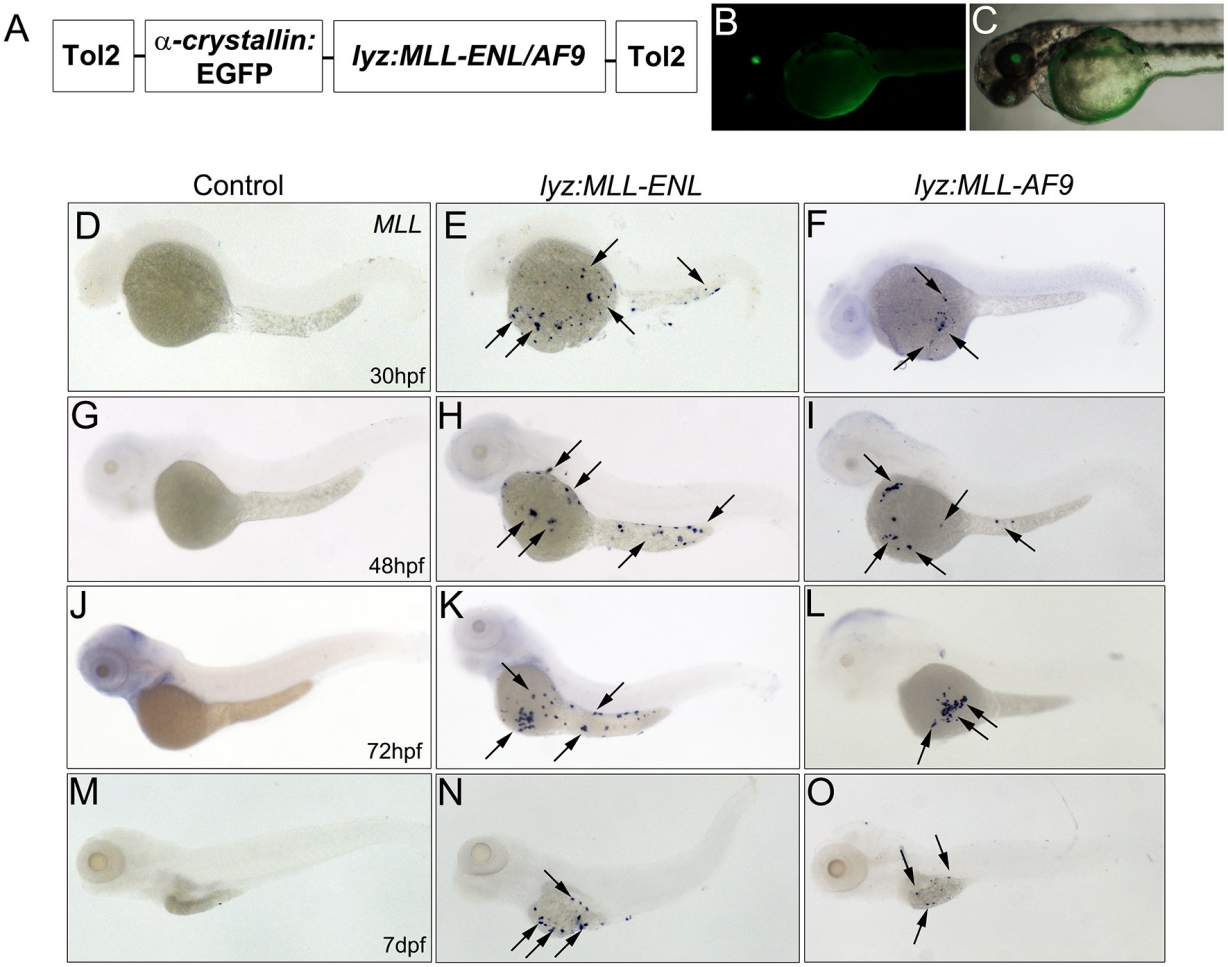
*MLL* is expressed on the yolk of *lyz*:*MLL-ENL* and *lyz*:*MLL-AF9* injected embryos. Diagram of the *lyz*:*MLL-ENL/AF9* construct used in the study (A). The α-crystallin promoter drives expression of EGFP in the lens of *lyz*:*MLL-ENL/AF9* injected embryos (B,C). *MLL* expression (all panels) was identified on the yolk of *lyz*:*MLL-ENL* and *lyz*:*MLL-AF9* embryos (arrows) but was absent from controls at 30 hpf (D-F), 48 hpf (G-I), 72 hpf (J-L) and 7 dpf (M-O).

Zebrafish have emerged as a powerful model system to study leukemogenesis by targeting known human fusion proteins in the myeloid or lymphoid lineage [21]. In particular, the fusion of the MLL (Lysine [K]-specific MethylTransferase 2A) gene to either ENL (MLLT1, Myeloid/Lymphoid or Mixed-Lineage Leukemia) or AF9 (MLLT3, Myeloid/Lymphoid or Mixed-Lineage Leukemia) are common rearrangements found in AML patients [3]. To determine if MLL-ENL or MLL-AF9 fusion proteins alter myeloid development in zebrafish, human FLAG-tagged MLL-ENL or FLAG-tagged MLL-AF9 were expressed under the zebrafish lysozyme C (*lyz*) promoter [27–29]. To confirm insertion of the construct into the genome, the final expression vector included the *α-crystallin* promoter driving EGFP expression in the lens (Fig 1A–1C) [30]. Similar to expression of other human fusion proteins, stable transgenic fish expressing *lyz*:*MLL-ENL* or *lyz*:*MLL-AF9* in the myeloid lineage did not survive, likely due to altered myelopoiesis inhibiting maturation [10]. Therefore, all analyses for this research were completed using transient transgenic embryos.

*Lyz* is expressed in myeloid progenitor cells, neutrophils, and macrophages during zebrafish development. Its expression is first detected on the yolk at 22 hours post fertilization (hpf), where it colocalizes with the myeloid progenitor marker, *spi1b*, on the yolk and in the intermediate cell mass (ICM) (S1 Fig) [27,29]. At 25 hpf, *lyz* positive cells migrate ventrally to the duct of Cuvier and enter into circulation to seed the peripheral blood island (PBI)/caudal hematopoietic tissue (CHT) [27]. Therefore, expression of *lyz*:*MLL-ENL* and *lyz*:*MLL-AF9* were analyzed at 30 hpf, 48 hpf, 72 hpf, and 7 days post fertilization (dpf) using a probe that detects human MLL (*MLL)*. Consistent with *lyz* expression in developing embryos, expression of *MLL* was observed on the yolk at 30 hpf (Fig 1E and 1F). Further analysis of *lyz*:*MLL-ENL* and *lyz*:*MLL-AF9* injected embryos at 48 hpf, 72 hpf, and 7 dpf identified expression of *MLL* exclusively on the yolk, often in clusters (Fig 1E, 1F, 1H, 1I, 1K, 1L, 1N and 1O). The absence of expression in uninjected embryos confirmed that the *MLL* probe did not cross react with endogenous zebrafish *mll* (Fig 1D, 1G, 1J and 1M). Given *lyz* is expressed in the ICM and *MLL* expression was observed exclusively on the yolk, we further validated the specificity of the *lyz* promoter. We expressed *mCherry* under the *lyz* promoter and identified mCherry+ cells in the CHT at 72 hpf (S2 Fig), confirming promoter specificity. Therefore, expression of *MLL-ENL* or *MLL-AF9* in the myeloid lineage induced the expansion of *MLL* expressing cells on the yolk of developing zebrafish embryos.

### Accumulation of MLL positive cells on the yolk is not due to cardiovascular defects

**Fig 2:**
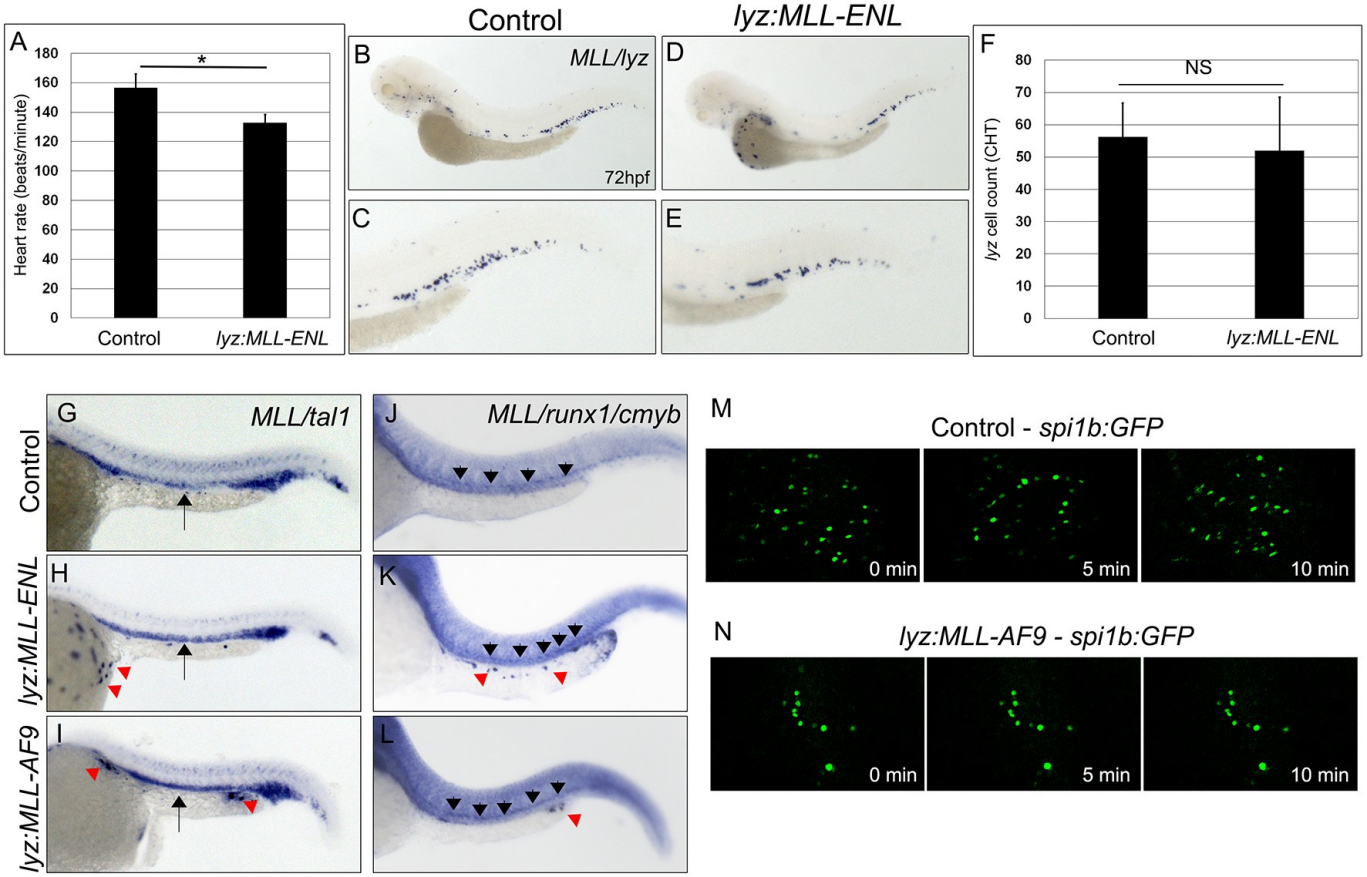
Accumulation of MLL positive cells on the yolk results from migration defects and not cardiovascular or hematopoietic defects. Average heart rates of control and *lyz*:*MLL-ENL* injected embryos at 48 hpf (A, N = 10). *MLL* (yolk) and *lyz* expression in control (B,C) and *MLL-ENL* expressing embryos (D,E). The number of *lyz* positive cells in the CHT in control and *lyz*:*MLL-ENL* injected embryos at 72 hpf (F, N = 12–18). MLL/Tal1 (G-I, arrows–*tal1*, red arrowheads-*MLL*, 24 hpf) and *MLL/runx1/cmyb* (J-L, black arrowheads-*runx1/cmyb*, red arrowheads-*MLL*, 36 hpf) expression were analyzed in control (G,J, N = 15–30), *lyz*:*MLL-ENL* (H,K, N = 13–30), and *lyz*:*MLL-AF9* (I,L, N = 17–25) embryos. Still images at 0, 5 and 10 minutes from *spi1b*:*GFP* timelapse videos for control (M) and lyz:MLL-AF9 (N) embryos. Statistical analysis was conducted using student’s t-test. * p<0.05. Anterior to the left.

*MLL* positive cells were exclusively found on the yolk in *lyz*:*MLL-ENL* expressing embryos, which is in contrast to wild type embryos, where *lyz* positive cells enter into circulation to seed the PBI/CHT [27]. To determine if *MLL*-positive cells remained on the yolk due to defects in cardiovascular development or blood flow, we analyzed heart rate, blood circulation, and endogenous *lyz* expression in *lyz*:*MLL-ENL* injected embryos. The heart rate was significantly, but not severely, decreased (133 bpm +/- 5.6) compared to uninjected control embryos (157 +/- 9.5) at 72 hpf (Fig 2A). However, this decrease in heart rate did not inhibit circulation of red blood cells at 48 hpf (S1 and S2 Videos) or *spi1b*:*GFP* positive cells at 72 hpf (S3 and S4 Videos) when examined in confirmed *MLL-ENL* expressing embryos. In addition, circulation was also confirmed by the expression of endogenous *lyz* positive cells in the CHT of *lyz*:*MLL-ENL* expressing embryos at 72 hpf (Fig 2B–2E), where the number of *lyz* positive cells was similar to uninjected controls. This result suggests that *lyz* positive cells that did not express *MLL-ENL* were able to seed the CHT (Fig 2F). Furthermore, we analyzed hematopoietic stem and progenitor cell (HSPC) markers, *tal1* and *runx1/cmyb*, in MLL-ENL and MLL-AF9 expressing embryos since blood flow and myeloid cell signaling are necessary for HSPC specification [31, 32]. *Tal1* and *runx1/cmyb* expression were normal in confirmed *lyz*:*MLL-ENL* and *lyz*:*MLL-AF9* expressing embryos compared to uninjected controls (Fig 2G–2L).

To better understand why MLL positive cells remained on the yolk, we employed timelapse imaging of *spi1b*:*GFP* embryos injected with *lyz*:*MLL-AF9*. Control *spi1b*:GFP positive cells were observed forming protrusions and rapidly migrating over the yolk at 48hpf (Fig 2M and S5 Video). In contrast, a subset of *spi1b*:*GFP* positive cells formed short protrusions and did not migrate but remained in the same location on the yolk in embryos injected with *lyz*:*MLL-AF9* (Fig 2N and S6 Video). Therefore, the accumulation of *MLL-ENL* and *MLL-AF9 expressing* cells on the yolk likely resulted from impaired migration and was not a result of defects in cardiovascular development.

### Lyz:MLL-ENL and lyz:MLL-AF9 positive cells express myeloid marker genes

**Fig 3:**
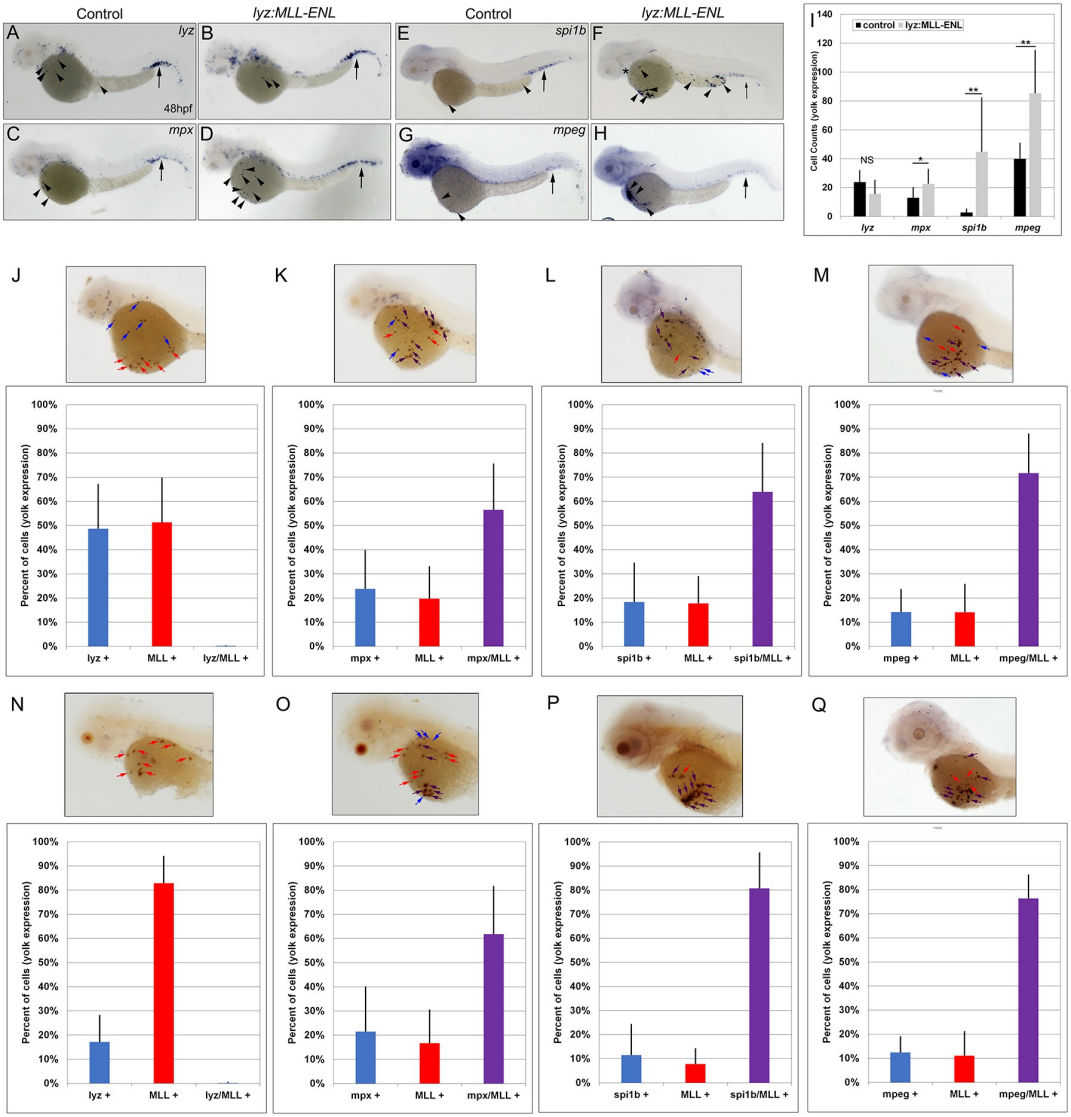
*MLL* expression colocalized with *mpx*, *spi1b*, and *mpeg* expression on the yolk of *lyz*:*MLL-ENL* injected embryos at 48 and 72 hpf. Expression of *lyz*, *mpx*, *spi1b*, and *mpeg* in control (A,C,E,G) and *lyz*:*MLL-ENL* injected (B,D,F,H) embryos at 48 hpf. Black arrows indicate expression in the CHT, arrowheads indicate expression on the yolk (A-H). The number of cells on the yolk expressing *lyz*, *mpx*, *spi1b*, and *mpeg* was assessed at 48 hpf (I, N = 10–46). *MLL* colocalization with *lyz* (0%), *mpx* (56.5% +/- 18.9%), *spi1b* (63.9% +/- 20.0%), and *mpeg* (71.4% +/- 16.1%) was determined at 48 hpf (J-M, N = 17–42) and with *lyz* (0%), *mpx* (61.8% +/- 19.7%), *spi1b* (80.7% +/- 14.7%), and *mpeg* (76.4% +/- 9.6%) at 72hpf (N-Q, N = 15–63). Blue, red, and purple bars indicate the percentage of cells stained on the yolk that were myeloid single positive (blue arrows), *MLL* single positive (red arrows), or myeloid marker/*MLL* double positive (purple arrows), respectively (J-Q). Statistical analysis was conducted using student’s t-test. * p<0.05, ** p<0.01, NS = not significant. Anterior to the left.

**Fig 4:**
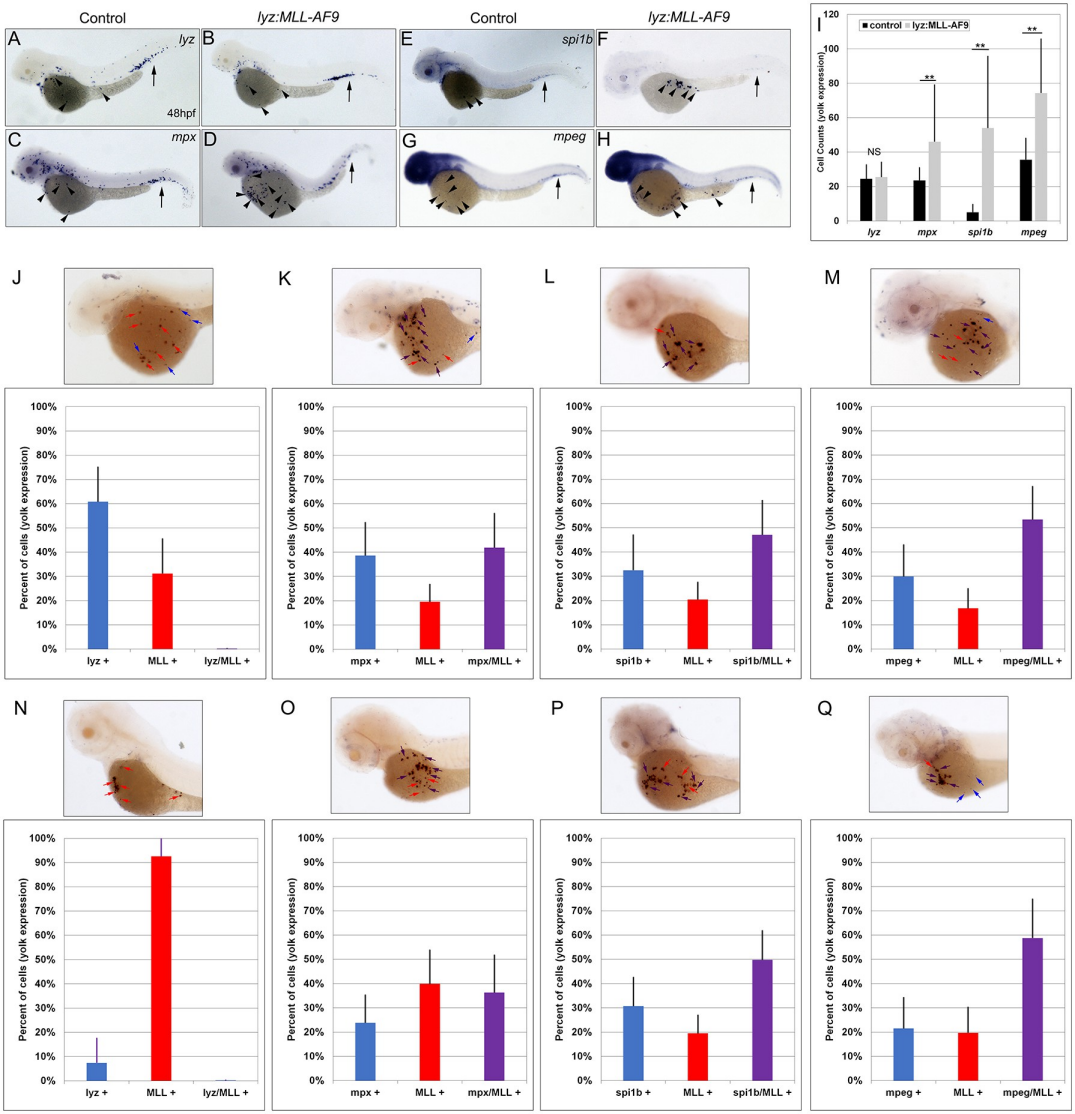
*MLL* expression colocalized with *mpx*, *spi1b*, and *mpeg* expression on the yolk of *lyz*:*MLL-AF9* injected embryos at 48 and 72 hpf. Expression of *lyz*, *mpx*, *spi1b*, and *mpeg* in control (A,C,E,G) and *lyz*:*MLL-AF9* injected (B,D,F,H) embryos at 48 hpf. Black arrows indicate expression in the CHT, arrowheads indicate expression on the yolk (A-H). The number of cells on the yolk expressing *lyz*, *mpx*, *spi1b*, and *mpeg* was assessed at 48 hpf (I, N = 31–62). *MLL* colocalization with *lyz* (0%), *mpx* (41.9% +/- 14.0%), *spi1b* (47.1% +/-14.1%), and *mpeg* (53.4% +/- 13.6%) was determined at 48 hpf (J-M, N = 37–52) and with *lyz* (0%), *mpx* (36.6% +/- 15.4%), *spi1b* (49.8% +/- 12.0%), and *mpeg* (58.8% +/- 16.0%) at 72hpf (N-Q, N = 26–34). Blue, red, and purple bars indicate the percentage of cells stained on the yolk that were myeloid single positive (blue arrow), *MLL* single positive (red arrow), or myeloid marker/*MLL* double positive (purple arrow), respectively (J-Q). Statistical analysis was conducted using student’s t-test. * p<0.05, ** p<0.01, NS = not significant. Anterior to the left.

To determine which myeloid cell-type expressed *MLL-ENL* and *MLL-AF9*, lineage analysis was completed using known markers for myeloid progenitor cells and macrophages (*spi1b*), neutrophils (*mpx*), neutrophils and macrophages (*lyz*), and macrophages (*mpeg*) at 48 and 72 hpf [27,29,33–36]. Although *MLL-ENL* and *MLL-AF9* are targeted by the *lyz* promoter to the myeloid lineage, expression of endogenous *lyz* was not expanded at 48 hpf (Figs 3A, 3B, 3I, 4A, 4B and 4I). In contrast, expression of *mpx*, *spi1b*, and *mpeg* were significantly increased on the yolk at 48 hpf (Figs 3C–3I and 4C–4I).

To confirm the results of single marker gene analyses (Figs 3I and 4I), double colorimetric whole mount *in situ* hybridization was completed on *lyz* targeted *MLL-ENL* and *MLL-AF9* expressing cells. The total number of stained cells on the yolk was counted and the percentage of cells that expressed the marker gene (*lyz*, *mpx*, *spi1b*, or *mpeg*), *MLL*, or both marker gene and *MLL* was calculated. Consistent with single probe analysis (Fig 3I), *MLL* did not colocalize with *lyz* at 48 or 72 hpf (Figs 3J, 3N, 4J and 4N). The percentage of stained cells on the yolk that expressed endogenous *lyz* decreased from ~55% at 48 hpf to ~12% at 72 hpf, while the percentage of stained cells that expressed *MLL* increased from ~40% to over 90% at the same timepoints in both *MLL-ENL* and *MLL-AF9* expressing embryos (Figs 3J, 3N, 4J and 4N). These results suggest that *lyz* expression is reduced or lost in cells that express *MLL-ENL* or *MLL-AF9*.

In contrast, *mpx*, *spi1b*, and *mpeg* expression colocalized with *MLL* on the yolk of *lyz*:*MLL-ENL* and *lyz*:*MLL-AF9* injected embryos at 48 and 72 hpf (Figs 3K–3M, 3O–3Q, 4K–4M and 4O–4Q). *MLL* colocalized with *mpx* in >40% of cells on the yolk at 48 and 72 hpf in *MLL-ENL* and *MLL-AF9* expressing embryos. Similarly, *spi1b* and *mpeg* colocalized with *MLL* in >50% of yolk cells analyzed at 48 and 72 hpf. Single positive *MLL* cells were visualized in all experiments, suggesting differential myeloid marker gene expression in cells expressing *MLL-ENL* or *MLL-AF9*. Taken together, these results demonstrate that expression of *MLL-ENL* and *MLL-AF9* in the myeloid lineage induced expansion of *mpx*, *spi1b*, and *mpeg* but not *lyz* expression on the yolk of zebrafish embryos.

### Increased expression of cdk9 and bcl2 in lyz:MLL-ENL and lyz:MLL-AF9 injected embryos

**Fig 5:**
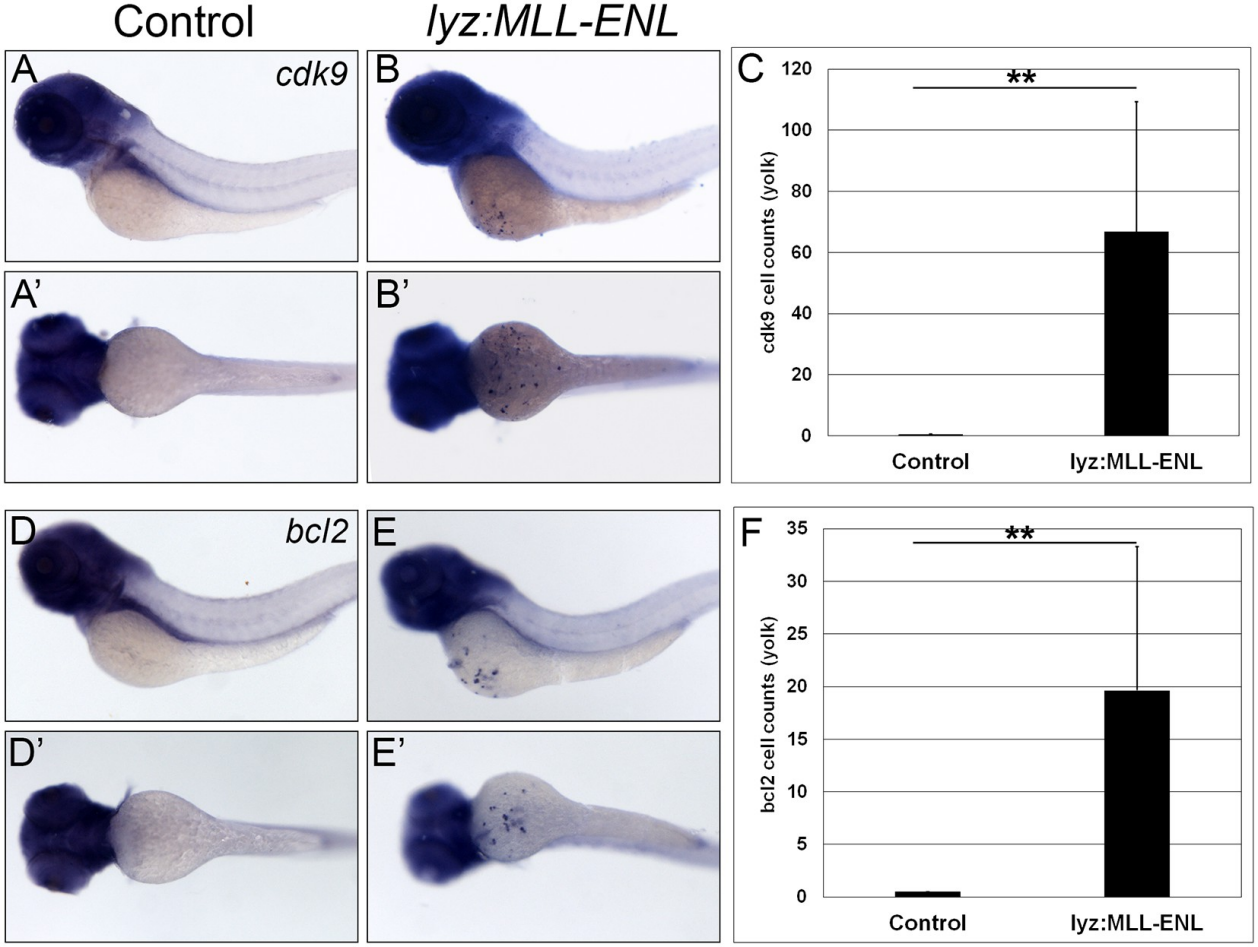
Myeloid targeted *MLL-ENL* induced expression of *cdk9 and bcl2 on the yolk*. *Cdk9* was expressed on the yolk of *lyz*:*MLL-ENL* injected embryos (B lateral; B’, ventral) compared to control embryos (A,A’). Quantitative analysis of *cdk9* positive cells on the yolk (C, N = 28). *Bcl2* expression was observed on the yolk of *lyz*:*MLL-ENL* injected embryos (E lateral; E’, ventral) compared to control embryos (D,D’). Quantitative analysis of *bcl2* positive cells on the yolk (F, N = 35). Statistical analysis was conducted using student’s t-test. ** p<0.01. Anterior to the left.

**Fig 6:**
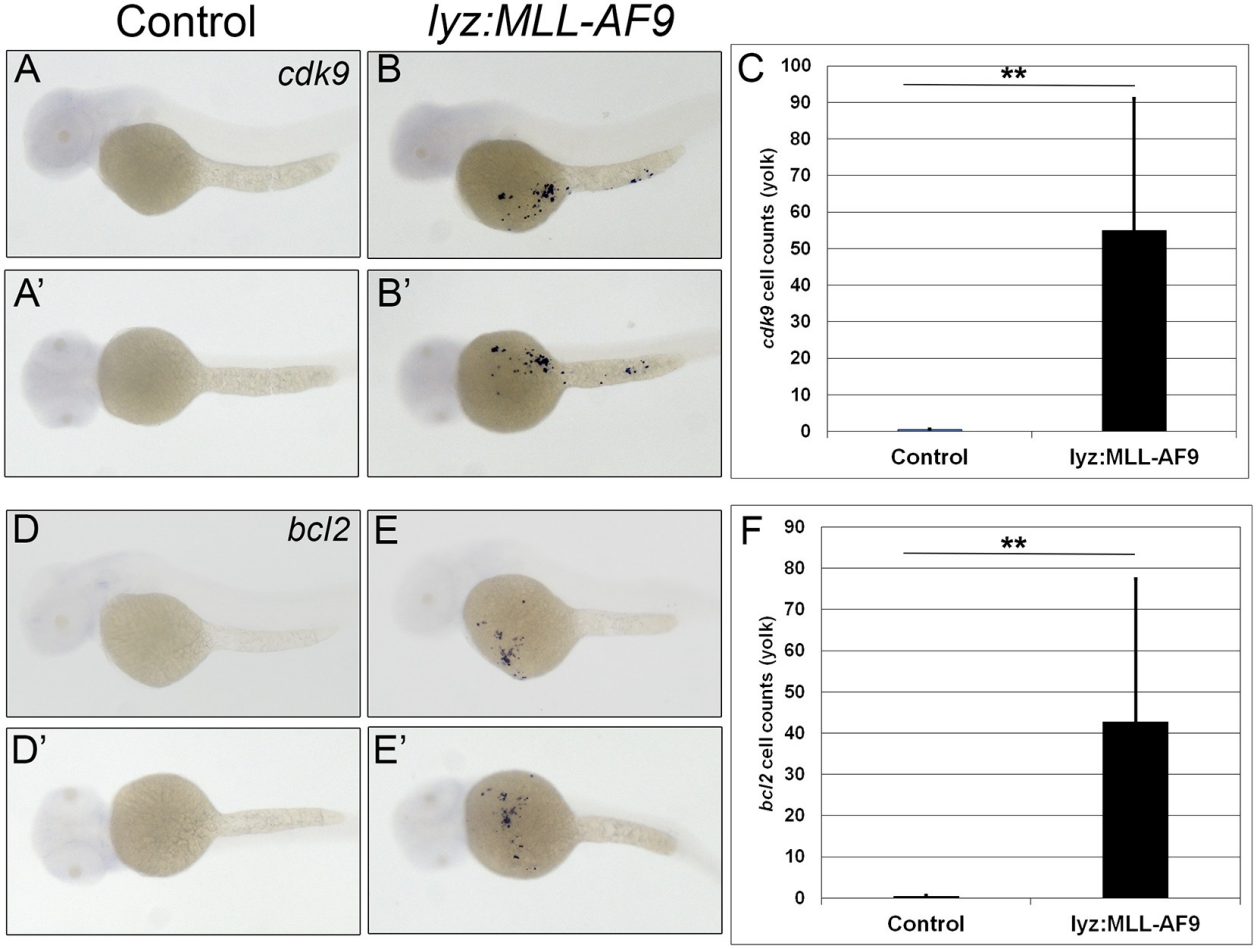
Myeloid targeted *MLL-AF9* induced expression of *cdk9 and bcl2 on the yolk*. *Cdk9* was expressed on the yolk of *lyz*:*MLL-AF9* injected embryos (B lateral; B’, ventral) compared to no expression on the yolk in control embryos (A,A’). Quantitative analysis of *cdk9* positive cells on the yolk (C, N = 31). *Bcl2* expression was observed on the yolk of *lyz*:*MLL-AF9* injected embryos (E lateral; E’, ventral) compared to control embryos (D,D’). Quantitative analysis of *bcl2* positive cells on the yolk (F, N = 30). Statistical analysis was conducted using students t-test. ** p<0.01. Anterior to the left.

Dysregulated transcription leads to proliferation of myeloblasts in patients with MLL-rearranged AML (MLL-r-AML). One key transcriptional regulator that is dysregulated is cyclin dependent kinase 9 (CDK9) [37,38]. CDK9 is a major component of the multiprotein positive transcription elongation factor b (PTEF-b) complex which is integral in promoting global transcription of target genes to enable cell survival [39]. Therefore, the expression of zebrafish *cdk9* was analyzed in control, *lyz*:*MLL*-ENL and *lyz*:*MLL-AF9* injected embryos. Consistent with mouse MLL-r models, *cdk9* expression increased from zero cells on the yolk of uninjected embryos to 66.8 (+/- 42.60) positive cells in *lyz*:*MLL-ENL* expressing embryos (Fig 5A–5C) and 50.0 (+/- 36.2) positive cells in *lyz*:*MLL-AF9* expressing embryos (Fig 6A–6C).

In addition to dysregulated transcription, inhibition of apoptosis through increased expression of pro-survival genes promotes cell survival and myeloid expansion in AML. One key gene that inhibits apoptosis in AML is B-cell lymphoma 2 (BCL2) [38,40,41]. Therefore, zebrafish *bcl2* expression was assessed in *lyz*:*MLL-ENL*, *lyz*:*MLL-AF9*, and control embryos. *Bcl2* expression on the yolk of *lyz*:*MLL-ENL* and *lyz*:*MLL-AF9* injected embryos increased from zero in control embryos to 19.6 (+/- 13.7) and 42.7 (+/- 34.7) positive cells, respectively (Figs 5D–5F and 6D–6F). Thus, expression of human MLL-ENL or MLL-AF9 in the myeloid lineage of zebrafish embryos leads to dramatically increased expression of endogenous *cdk9* and *bcl2*, similar to mouse models and patients with MLL-r-AML [37,42].

### Dose dependent effects of Venetoclax and Flavopiridol on myelopoiesis in zebrafish embryos

**Fig 7:**
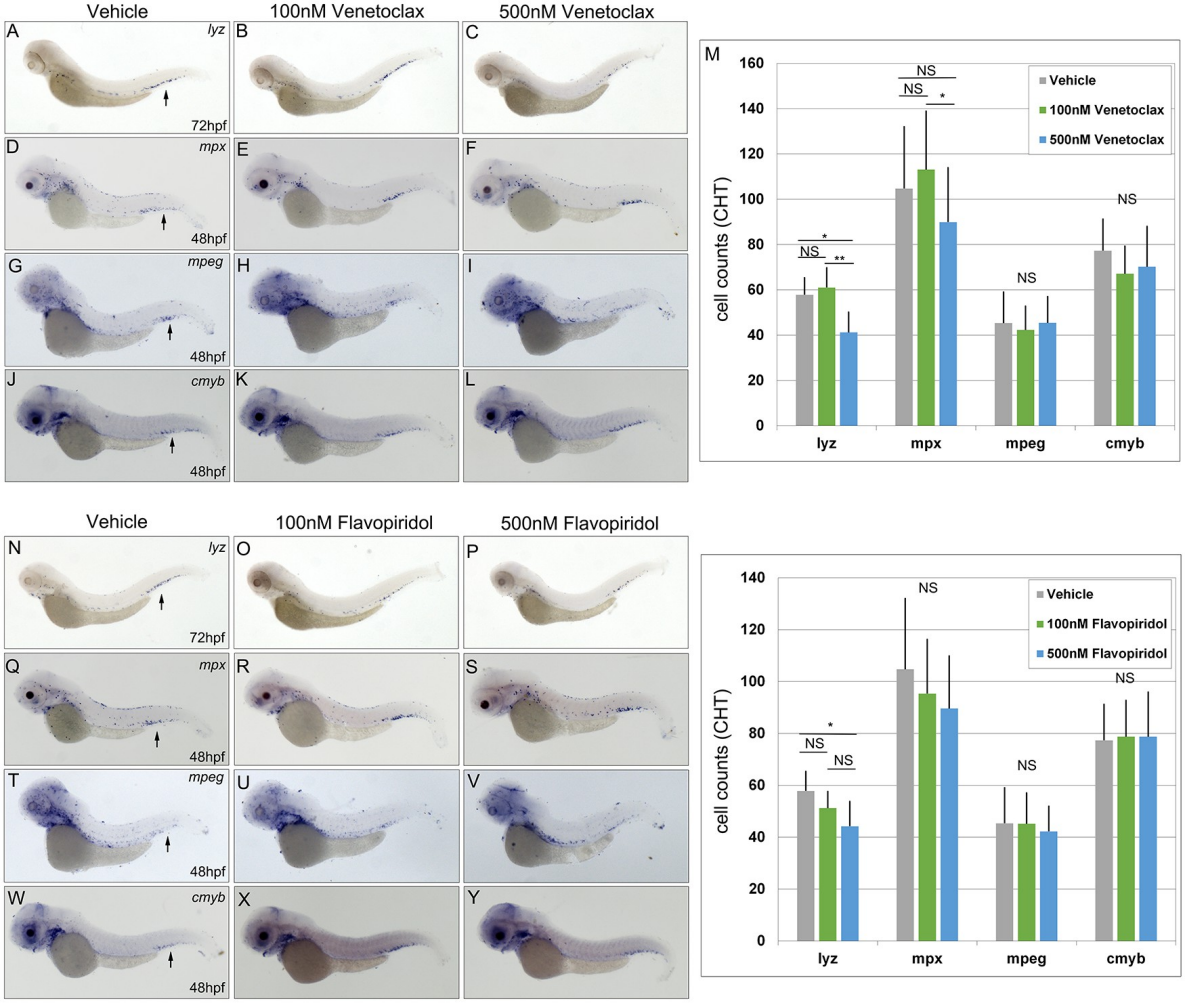
Dose dependent effect of Venetoclax and Flavopiridol on myelopoiesis. Embryos were treated with 100nM or 500nM of Venetoclax (A-M) or 100nM and 500nM Flavopiridol (N-Z) and assessed for expression of *lyz* (A-C, N-P, N = 10), *mpx* (D-F, Q-S, N = 16–19), *mpeg* (G-I, T-V, N = 20), and *cmyb* (J-L, W-Y, N = 16–20) by counting the number of positive cells expressing each marker in the CHT (M,Z). Arrows indicate marker gene expression in the CHT. Statistical analysis was conducted using one-way ANOVA followed by Tukey HSD. * p<0.05, ** p<0.01, NS = not significant. Anterior to the left.

To assess the effects of dysregulated *cdk9* and *bcl2* expression on zebrafish embryos, pharmacological inhibitors of these two target proteins were investigated. The BCL2 inhibitor, Venetoclax, was selected because it is a small-molecule selective inhibitor that is currently approved to treat patients with chronic lymphocytic leukemia (CLL), small lymphocytic leukemia (SLL), and newly diagnosed AML in combination with other therapies [43]. Control embryos were treated with 100 and 500nM of Venetoclax beginning at 24 hpf and assessed for expression of myeloid markers (*lyz*, *mpx*, *mpeg*) and the myeloid and hematopoietic stem cell marker, *cmyb* (Fig 7A–7L). Expression of *lyz* and *mpx* were unchanged in embryos treated with 100nM of Venetoclax, but were significantly decreased at 500nM (Fig 7M). Hematopoietic stem cells in the CHT were assessed by *cmyb* expression at 48 hpf and found to be unaffected at 100nM and 500nM concentrations compared to control embryos (Fig 7M). Importantly, inhibition of Bcl2 at 100nM or 500nM did not cause any visible gross morphological defects at 48 or 72 hpf. Therefore, inhibition of Bcl2 using 500nM of Venetoclax inhibits myeloid but not hematopoietic development.

To determine the effects of Cdk9 inhibition on zebrafish development, a small-molecule inhibitor, Flavopiridol, was employed. Flavopiridol is a flavonoid alkaloid inhibitor that binds to the ATP-binding site of CDKs, reversibly inhibiting their function. It has been evaluated in clinical trials to treat AML in combination with other pharmacological inhibitors [44]. Treatment of zebrafish embryos beginning at 24 hpf with 100nM or 500nM Flavopiridol did not cause any gross morphological defects at 48 or 72 hpf (Fig 7N–7Z). These concentrations are significantly less than those tested previously in zebrafish embryos, wherein 5μM caused edema and a curved body axis [45]. Embryos incubated with 100nM Flavopiridol had similar numbers of *lyz*, *mpx*, *mpeg*, and *cmyb* positive cells in the CHT compared to control embryos. Treatment at 500nM caused a significant decrease in *lyz* positive cells, but did not affect *mpx*, *mpeg*, or *cmyb* positive cells in the CHT (Fig 7Z). Therefore, treatment of embryos with 500nM Flavopiridol reduced the number of *lyz* positive but not *mpx* positive myeloid cells or *cmyb* positive hematopoietic stem cells in the CHT.

### Combinatorial treatment with Venetoclax and Flavopiridol partially recovers the myeloid expansion observed in lyz:MLL-ENL and lyz:MLL-AF9 expressing embryos

**Fig 8:**
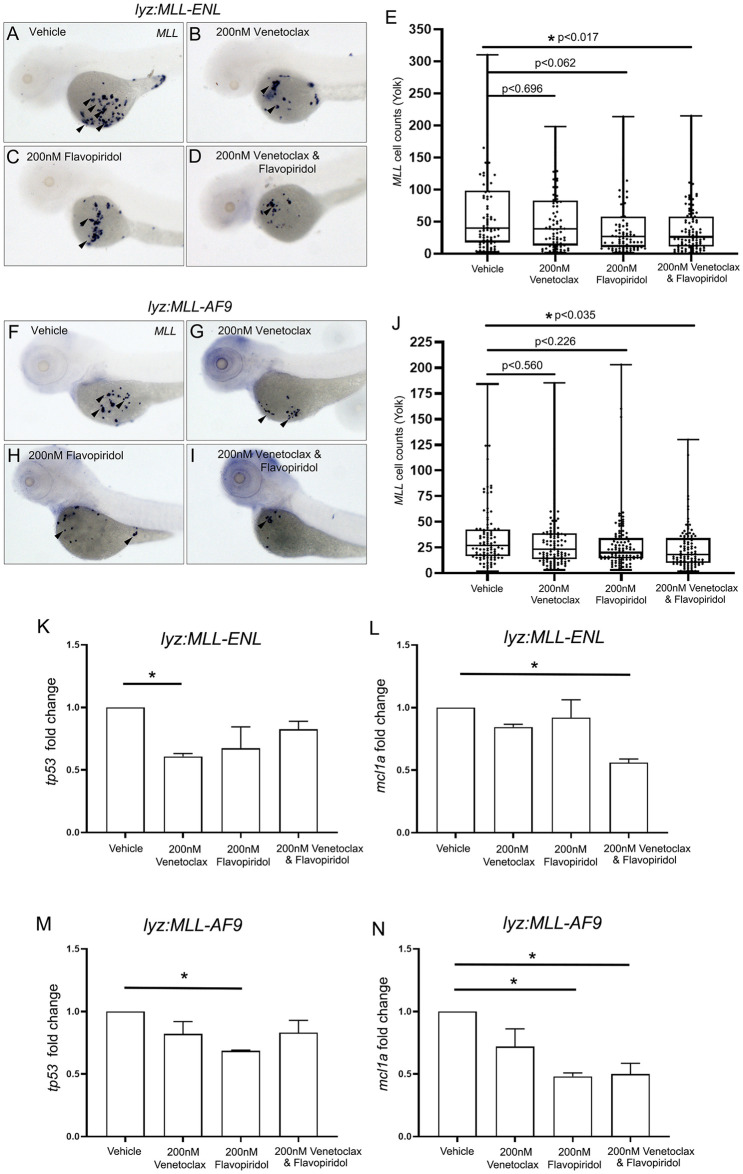
Venetoclax and Flavopiridol co-treatment significantly reduced *MLL* expression on the yolk of *lyz*:*MLL-ENL* and *lyz*:*MLL-AF9* injected embryos. *Lyz*:*MLL-ENL* injected embryos were incubated with 200nM DMSO (A, N = 82), 200nM Venetoclax (B, N = 76), 200nM Flavopiridol (C, N = 84) or 200nM Venetoclax and Flavopiridol (D, N = 98) at 24 hpf and the number of *MLL* positive cells was assessed at 72 hpf (E). *Lyz*:*MLL-AF9* injected embryos were incubated with 200nM DMSO (F, N = 96), 200nM Venetoclax (G, N = 105), 200nM Flavopiridol (H, N = 102) or 200nM Venetoclax and Flavopiridol (I, N = 95) at 24 hpf and the number of *MLL* positive cells was assessed at 72 hpf (E,J). *Lyz*:*MLL-ENL* (K,L) and *lyz*:*MLL-AF9* (M,N) injected embryos were assessed for expression of *tp53* and *mcl1a* by qPCR (N = 2). Statistical analysis was conducted using one-way ANOVA followed by Tukey HSD. * p<0.05. Anterior to the left.

To determine if *cdk9* and *bcl2* promotes myeloid expansion in *lyz*:*MLL-ENL* and *lyz*:*MLL-AF9* injected embryos, Venetoclax and Flavopiridol were investigated individually and in combination. Each drug was first assessed at 200nM in wild type embryos (S3 Fig) to determine its effect on myeloid development in control embryos. Treatment of embryos beginning at 24 hpf showed no significant change in the number of *lyz* positive cells in Venetoclax or combinatorially treated embryos, but did show a significant reduction for Flavopiridol alone when assessed at 72 hpf (S3 Fig). Furthermore, gross morphological defects were not observed in embryos treated with either drug alone or in combination. Therefore, 200nM Venetoclax or Flavopiridol alone or in combination were used in *lyz*:*MLL-ENL and lyz*:*MLL-AF9* injected embryos. To assay rescue of myeloid expansion, *MLL* positive cells were counted on the yolk at 72 hpf. Vehicle treated *MLL-ENL* embryos averaged 61.4 (+/- 56.4) and vehicle treated *MLL-AF9* embryos averaged 35.1 (+/- 29.3) *MLL* positive cells per embryo, while Venetoclax and Flavopiridol alone averaged 53.5 (+/- 49.5) and 42.9 (+/- 44.2) positive cells in *MLL-ENL* embryos and 30.3 (+/- 25.7) and 27.9 (+/- 28.9) in *MLL-AF9* embryos, respectively (Fig 8A–8J). Neither drug treatment alone caused a statistically significant reduction in *MLL* expressing cells. However, treatment with a combination of Venetoclax and Flavopiridol significantly reduced the number of *MLL* positive cells on the yolk to 40.2 (+/- 40.8) *in lyz*:*MLL-ENL* injected embryos and 24.7 (+/- 21.5) in *lyz*:*MLL-AF9* injected embryos (Fig 8E and 8J). Thus, combinatorial treatment of lyz:MLL-ENL and lyz:MLL-AF9 embryos partially recovered the myeloid expansion observed on the yolk.

To further characterize the reduction of MLL positive cells in Venetoclax and Flavopiridol co-treated embryos, we analyzed levels of the proapoptotic gene, *tp53*, and the pro-survival gene, *mcl1a*. Treatment of *lyz-MLL-ENL* embryos with Venetoclax or *lyz*:*MLL-AF9* embryos with Flavopiridol induced a significant reduction in endogenous *tp53* expression compared to vehicle treatment alone, while the remaining treatment groups did not show a significant change in *tp53*. These results suggest that the reduction in *MLL* positive cells occurs in a Tp53-independent manner (Fig 8K and 8M). This is consistent with previous reports that demonstrate Venetoclax and Flavopiridol induced cell death independent of TP53 in leukemia cell lines [46,47]. Mcl-1 is a member of the Bcl-2 family of prosurvival genes and is often overexpressed in AML [48,49]. Venetoclax is thought to increase the binding affinity of Mcl-1 to proapoptotic proteins, such as Bim. Therefore, Venetoclax treatment alone is reported to have a limited effect on cancers that have elevated Mcl-1 [50]. Flavopiridol inhibits pTEFb activation resulting in down-regulation of short-lived proteins, such as Mcl-1, in the absence of active transcription [51]. Thus, Flavopiridol increases the sensitivity of Venetoclax in Mcl-1 elevated leukemia [38,49,52,53]. Co-treatment with Flavopiridol and Venetoclax in *lyz*:*MLL-ENL* and *lyz*:*MLL-AF9* expressing embryos significantly reduced the level of *mcl1a*, consistent with AML models. Therefore, inhibition of Bcl2 and Cdk9 reduces *mcl1a* expression leading to a partial recovery of the myeloid expansion observed in *lyz*:*MLL-ENL and lyz*:*MLL-AF9* injected embryos.

## Discussion

MLL rearrangements (MLL-r) occur in approximately 10% of AML patients, with 34% of those harboring an MLL-ENL or MLL-AF9 rearrangement. Despite modern therapies, patients with MLL-r-AML have poor outcomes compared to those with non-MLL-r-AML [54]. Therefore, generating new animal models is critical to identify novel drug treatments to improve patient outcomes. Our results demonstrated that expression of *MLL-ENL* or *MLL-AF9* in the myeloid lineage caused an expansion of myeloid cells and increased expression of endogenous *cdk9* and *bcl2* on the yolk of zebrafish embryos. *MLL-ENL* expressing embryos co-treated with the CDK9 inhibitor, Flavopiridol, and BCL2 inhibitor, Venetoclax, significantly reduced the number of *MLL* positive cells compared to vehicle and individual treatments alone. Such results are consistent with previous reports indicating that other CDK9 inhibitors, by inhibiting the pTEFb transcription elongation factor, can increase the anti-tumor effects of Venetoclax AML models [55]. These results further support the use of zebrafish to recapitulate AML phenotypes and identify novel treatments for therapeutic intervention.

Stable lines of *lyz*:*MLL-ENL* and *lyz*:*MLL-AF9* were sought using the Tol2-mediated transgenesis system [6] but all fish that were raised lacked stable germline insertion in the myeloid lineage. Our inability to raise stable lines could be a result of embryonic myeloid and hematopoietic deficiencies that resulted from MLL-r insertion. Therefore, all analysis for this research focused on transient transgenic embryos.

Primitive myeloid cells arise from the lateral plate mesoderm (LPM) derived rostral blood island (RBI) and the inner cell mass (ICM) during zebrafish development [11,12]. *Spi1b* positive cells migrate to the anterior yolk and begin to express *lyz* at approximately 22 hpf. At the onset of blood flow, a subset of *lyz* positive cells then enter circulation to populate the ICM [11,27–29,33]. We identified single positive *lyz* and *spi1b* cells as well as double positive *lyz* and *spi1b* cells on the yolk, while all *lyz* positive cells in the ICM co-expressed *spi1b* in control embryos at 24 hpf [27,29]. In our model, *MLL* expression was only found on the yolk and not identified in the ICM of *lyz*:*MLL-ENL* injected embryos. Furthermore, *lyz* positive cells that did not express MLL-ENL were able to enter circulation to populate the ICM. Timelapse imaging of *spi1b*:*GFP* positive cells in *lyz*:*MLL-AF9* injected embryos suggested reduced migration inhibited these cells from entering circulation and induced the *MLL* expansion observed on the yolk in transgenic embryos. The absence of *lyz* colocalization with *MLL* was unexpected given the *lyz* promoter drove expression of both *MLL-ENL* and *MLL-AF9*. This result suggested that *lyz* expression was reduced or lost in these cells possibly pointing to a role for MLL-ENL and MLL-AF9 in regulating the transcription or stability of *lyz* transcripts. We also observed an increase in the number of *spi1b* and *mpx* positive cells on the yolk which was consistent with previous models [10,18,23,24,26]. The increase in *mpeg* positive cells in lyz:MLL-ENL and lyz:MLL-AF9 expressing embryos suggested that these cells belong to the monocytic lineage. *However*, *MLL* differentially colocalized with *spi1b*, *mpx*, and *mpeg* markers in both MLL-ENL and MLL-AF9 expressing embryos and the presence of single positive *MLL* cells in all embryos examined suggested variable myeloid cell maturation. Our results are similar, but not identical, to previous research in zebrafish embryos where ectopically expressed *MLL-AF9* mRNA increased expression of myeloid markers including *lcp1*, *mpx*, *spi1b*, and *lyz*, as well as the hematopoietic markers, *runx1* and *cmyb* [26]. Our results are likely different due to targeted expression of *MLL-ENL* and *MLL-AF9* to the myeloid lineage as opposed to ubiquitous expression using mRNA.

MLL-r-AML is characterized by dysregulated cell proliferation and reduced apoptosis. BCL2 is a key survival gene that is overexpressed in both AML patients and cell lines [42]. BCL2 sequesters proapoptotic proteins, such as BIM, to inhibit cell death and arrest cells in G0 [56,57]. We determined that expression of *MLL-ENL* or *MLL-AF9* in myeloid cells resulted in increased expression of endogenous *bcl2*, similar to AML patients. Increased expression of *bcl2* can be a consequence of dysregulated CDK9 which leads to altered transcriptional elongation and mRNA maturation of target genes, including MCL-1 [37]. Zebrafish embryos expressing *lyz*:*MLL-ENL* and *lyz*:*MLL-AF9* exhibited increased expression of *cdk9* compared to controls. The observed increase in *bcl2* and *cdk9* was exclusively on the yolk, consistent with human *MLL* expression in *MLL-ENL* or *MLL-AF9* expressing embryos. To determine if Bcl2 and Cdk9 inhibition alters normal embryonic development we employed Venetoclax (ABT-1099) and Flavopiridol, respectively. Venetoclax inhibits the interaction between BCL2 and BIM, allowing BIM to initiate apoptosis [42,43,56]. Flavopiridol is a CDK family pharmacological inhibitor that preferentially targets CDK9 by binding to the ATP site inhibiting protein activity and preventing transcription of target genes such as MCL-1, which confers resistance to Venetoclax [38,44,58,59]. We used low concentrations that were physiologically relevant to humans since previously it was shown that higher doses (1-5uM) of Flavopiridol caused morphological defects in zebrafish embryos, including tail curvature and edema [45]. Wild type zebrafish embryos treated with low concentrations of Venetoclax and Flavopiridol were morphologically normal with only a significant reduction in *lyz* positive cells at 500nM of either drug and a significant reduction in *mpx* positive cells with 500nM Venetoclax. Similar to findings in AML cell lines, we observed a significant reduction in the number of *MLL* positive cells in *MLL-ENL* and MLL-AF9 expressing embryos co-incubated with Venetoclax and Flavopiridol compared to embryos treated with vehicle or either drug alone. In addition, co-treatment of *MLL-ENL* and *MLL-AF9* embryos with Venetoclax and Flavopiridol significantly decreased endogenous *mcl1a* expression, consistent with AML patients and cell culture. Taken together, our results demonstrated that expression of human *MLL-ENL* or MLL-AF9 in the myeloid lineage of zebrafish embryos induced expression of *bcl2* and *cdk9*, leading to myeloid cell expansion, similar to patients with MLL-r-AML.

Future research will seek to use additional pharmacological inhibitors to determine if synergistic treatments improve outcomes without negatively affecting endogenous myeloid cell development and maintenance. Thus, our results further support the use of zebrafish as a valuable preclinical model for hematological malignancies and will be advantageous in identifying novel targets for drug discovery.

## Materials and methods

### Ethics statement

Wild type (AB, WIK), *Tg(mpeg1*.*1*:*dendra2)*^*umw12Tg*^, Tg(mpx:dendra)^umw4Tg^ [60] and Tg(Gal4:sp1b;UAS:EGFP) [61] lines were raised as previously described [62–65] under approved IACUC protocols, and in compliance with International Animal Care and Use Committee (IACUC) and American Veterinary Medical Association (AVMA) guidelines. Fish were not selected based on sex and no randomization was used in this study.

### *Lyz*:*MLL-ENL* and *lyz*:*MLL-AF9* expression vector synthesis and injections

Human FLAG tagged *MLL-ENL* (pMSCV-FlagMLL-pl-ENL) and *MLL-AF9* (pMIG-FLAG-MLL-AF9) were purchased from Addgene (plasmid #20873 and #71443 respectively). *MLL-ENL* was cloned into the pME vector (Invitrogen) using EcoRI and MLL-AF9 was cloned into the pME vector using XhoI. Directionality was confirmed using sequencing. Gateway recombination technology (Invitrogen) was used to generate the *lyz* targeted *MLL-ENL* and *MLL-AF9* vectors. The destination vector contained *α-crystallin*:*EGFP* which drives enhanced green fluorescent protein (*EGFP*) expression in the lens of zebrafish embryos beginning at 2 dpf. Embryos were injected at the one cell stage with 25 nanograms (ng) of *lyz*:*MLL-ENL* or *lyz*:*MLL-AF9* and 25 ng of transposase mRNA, and raised under standard conditions.

### In situ hybridization

Digoxigenin-labeled antisense riboprobes were synthesized with either T7, T3, or Sp6 RNA polymerase from cloned cDNAs and subsequently hybridized with zebrafish embryos, as previously described [62–64,66]. *Myb* and *mpx* were generated as previously described [11,67]. *MLL*, *mpeg*, *lyz*, *spi1b*, *bcl2* and *cdk9* were amplified (S1 Table), cloned into the pSCA vector (Agilent), sequenced, and probes generated as previously described [63,66,67].

Double colorimetric WISH was completed using fluorescein labeled *MLL* riboprobe that was synthesized as described above. Fluorescein-labeled *MLL* and digoxigenin-labeled *mpx*, *spi1b*, *mpeg*, *lyz*, *tal1*, *runx1*, and *cmyb* probes were hybridized to embryos and developed as previously described [68]. Embryos were mounted in 3% methyl cellulose and imaged using a Nikon AZ100 multi-zoom fluorescent stereo microscope using Elements 4.50 software.

### Venetoclax and Flavopiridol treatment

Embryos were dechorionated and resuspended in 1 mL of 1X E3 media with 0.004% 1-phenyl-2-thiourea (PTU) and incubated with Venetoclax or Flavopiridol (MedChem Express) at the desired drug concentration in 12-well dishes beginning at 24 hpf. Dimethyl sulfoxide (DMSO) was used to solubilize the drugs and was also used as a vehicle for control experiments. Drug treatments were continuous at 28° C until 72 hpf when embryos were fixed in 4% PFA/PBS.

For recovery experiments, *lyz*:*MLL-ENL* injected embryos were dechorionated and split evenly in a 12 well dish. Embryos were then incubated in 1X E3 media and 0.004% PTU containing either 200 nM Venetoclax, 200 nM Flavopiridol, 200 nM Venetoclax and Flavopiridol, or DMSO beginning at 24 hpf at 28° C before being fixed in 4% PFA/PBS at 72 hpf.

### Quantitative PCR

Embryos were collected from lyz:MLL-ENL and lyz:MLL-AF9 embryos treated with DMSO, 200nM Venetoclax, 200nM Flavopiridol, or 200nM Venetoclax and Flavopiridol beginning at 24 hpf until 60hpf, dechorionated, and total RNA was extracted as previously described [64,66]. Concentration and purity of total RNA was assessed using a Nanodrop 1000 spectrophotometer (Thermo Fisher Scientific). cDNA was prepared as previously described using approximately 1μg of RNA per reaction [64,66]. qPCR primers for *mcl1a* and *tp53* were previously validated (S1 Table).

All reactions were performed in triplicate using 2x SensiFAST SYBR No-ROX Mix (Bioline, BIO-98005). qPCR analysis was performed using a micPCR system (Bio Molecular Systems, M0000636). Single product amplification was confirmed using both a melt curve and direct sequencing of the product. Gene expression fold change was analyzed using the 2^-ΔΔC^_T_ method and was normalized to elongation-factor 1-alpha (ef1α) [69].

### Video microscopy

Live embryos were imaged using differential interference contrast optics or epifluorescence after transient anesthesia and immobilization on a Nikon AZ100 multi-zoom fluorescent stereo microscope using Elements 4.50 software. For each condition, embryos were imaged, individually fixed, and analyzed for *MLL* expression. 5–10 embryos were analyzed per condition. Timelapse videos of *Spi1b*:*GFP* embryos were imaged every thirty seconds for ten minutes on a Nikon C2 laser scanning confocal on a Nikon Eclipse Ni microscope using Elements AR 4.50 software.

### Statistical analysis

Statistical analyses were conducted using one-way ANOVA followed by Tukey’s HSD for pharmacological inhibitor treatments and qPCR. Statistical analysis was conducted using Student’s t-test for marker gene analysis and heart rates. Center values are calculated as the mean and error bars are standard deviations.

## Supporting information

S1 Fig *Lyz* and *spi1b* colocalize on the yolk and ICM at 24hpf. Localization of *spi1b* (blue arrowheads), *lyz* (red arrowheads), and *spi1b* and *lyz* (purple arrowheads) on the yolk ball (A) and ICM (B) at 24hpf. Anterior to the left.
(TIF)

S2 Fig *Lyz*:*mCherry* injected zebrafish embryos. mCherry positive cells are observed in zebrafish embryos at 72hpf in transient transgenic *lyz:mCherry* embryos (A). mCherry positive cells were identified in the CHT (B), consistent with endogenous *lyz* expression.
(TIF)

S3 Fig Venetoclax and Flavopiridol effect on *lyz* expression. Wild type embryos treated with DMSO, 200nM Venetoclax, 200nM Flavopiridol, or 200nM Venetoclax and Flavopiridol were assessed for the number of *lyz* positive cells in the CHT at 72 hpf. N = 10. *P<0.05.
(TIF)

S1 Table Primers used in this study. (PDF)

S1 Raw Data Index of supplementary data tables for this study. (XLSX)

S1 Video Red blood cells circulate in wild type embryos at 48hpf. (AVI)

S2 Video Red blood cells circulate in *lyz*:*MLL-ENL* injected embryos. (AVI)

S3 Video *Spi1b*:*GFP* positive cells circulate in wild type embryos. (AVI)

S4 Video *Sp1b*:*GFP* positive cells circulate in *MLL-ENL* injected embryos. (AVI)

S5 Video *Spi1b*:*GFP* positive control cell migration on the yolk at 48hpf. (AVI)

S6 Video *Spi1b*:*GFP* positive *lyz*:*MLL-AF9* cell migration on the yolk at 48hpf. (AVI)
