# Supplementary figures and images for: Myeloid Targeted Human MLL-ENL and MLL-AF9 Induces cdk9 and bcl2 Expression in Zebrafish Embryos
Source: PLoS Genet. 2024 Jun 3;20(6):e1011308. doi: 10.1371/journal.pgen.1011308 (PMC11175583; doi:10.1371/journal.pgen.1011308)

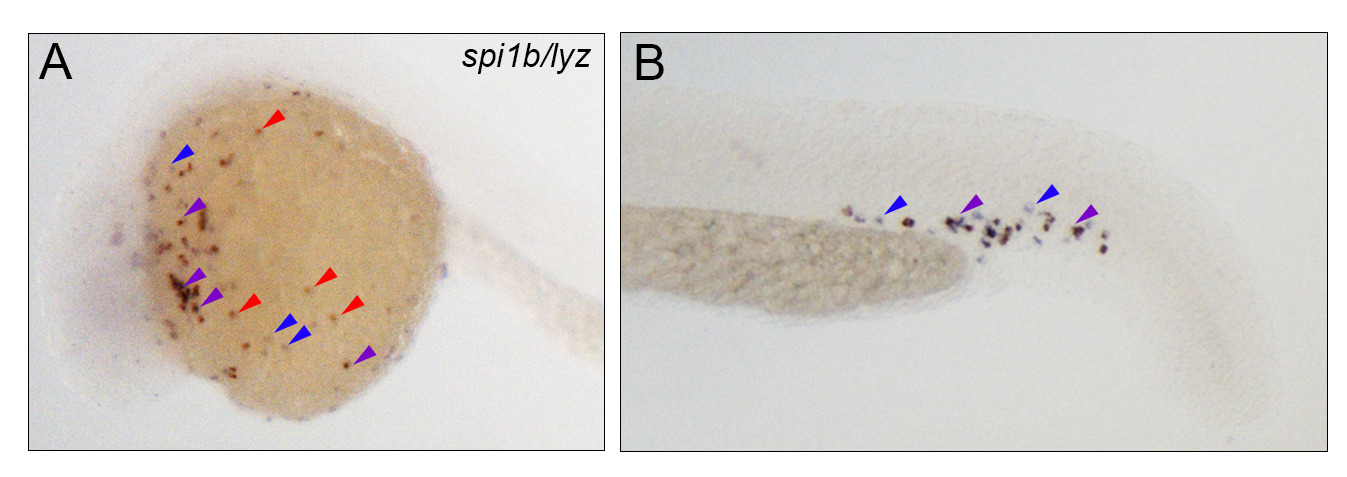

Supplement: S1 Fig — Localization of spi1b (blue arrowheads), lyz (red arrowheads), and spi1b and lyz (purple arrowheads) on the yolk ball (A) and ICM (B) at 24hpf. Anterior to the left. (TIF) [file pgen.1011308.s001.tif]

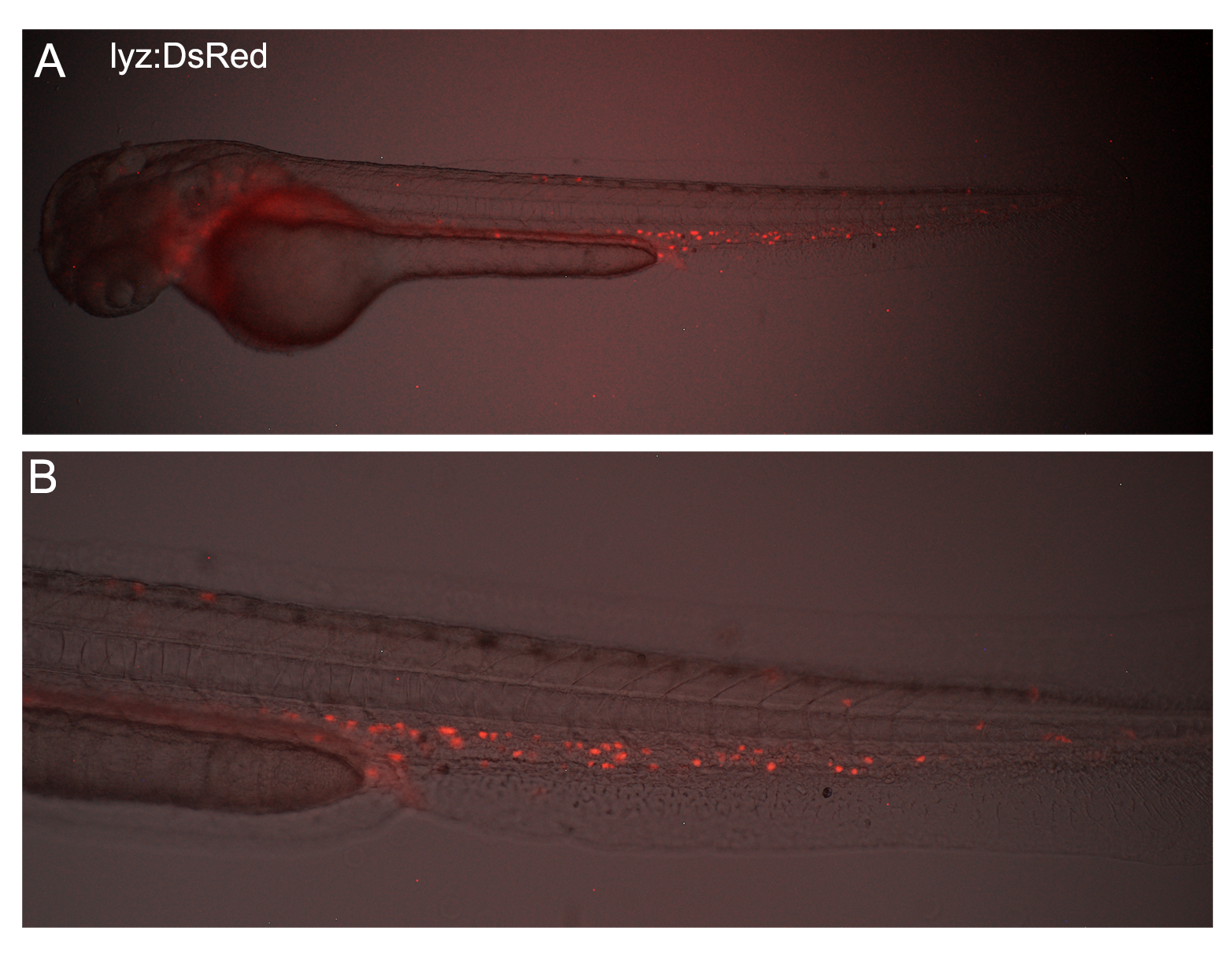

Supplement: S2 Fig — mCherry positive cells are observed in zebrafish embryos at 72hpf in transient transgenic lyz:mCherry embryos (A). mCherry positive cells were identified in the CHT (B), consistent with endogenous lyz expression. (TIF) [file pgen.1011308.s002.tif]

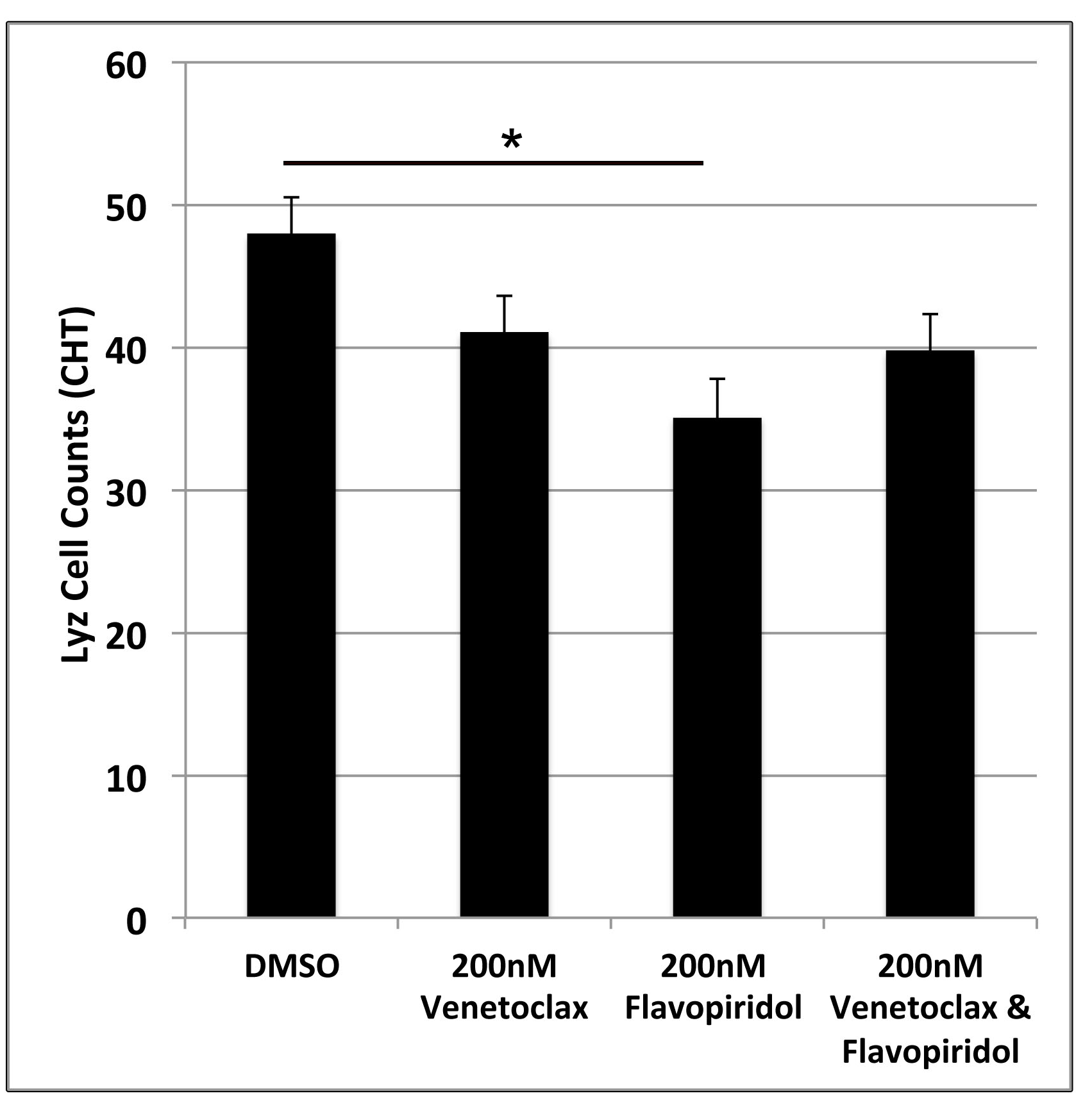

Supplement: S3 Fig — Wild type embryos treated with DMSO, 200nM Venetoclax, 200nM Flavopiridol, or 200nM Venetoclax and Flavopiridol were assessed for the number of lyz positive cells in the CHT at 72 hpf. N = 10. *P<0.05. (TIF) [file pgen.1011308.s003.tif]
