# Supplementary material for: Myeloid Targeted Human MLL-ENL and MLL-AF9 Induces cdk9 and bcl2 Expression in Zebrafish Embryos
Source: PLoS Genet. 2024 Jun 3;20(6):e1011308. doi: 10.1371/journal.pgen.1011308 (PMC11175583; doi:10.1371/journal.pgen.1011308)
Supplement: S1 Table — (PDF) [file pgen.1011308.s004.pdf]

Gene	Primer sequence (5' – 3')
Forward *bcl2*	GACAATCGGAATATTGTGGAG
Reverse *bcl2*	GTATGAAAACGGGTGGAACAC
Forward *cdk9*	CAGCCAATGTCCTGATCACC
Reverse *cdk9*	CCAGAAGAAGTCGTGGTTGAGAG
Forward *mpeg*	GTTACAGCACGGGTTCAAGTCC
Reverse *mpeg*	GCTGCTTTACGCACTGTTGAAG
Forward *lyz*	GAGGCTGGCAGTGGTGTTTTTGTG
Reverse *lyz*	GGTAATCAGGCTCGGAGGCTTTG
Forward *spi1b*	CAGAATGGAGGGGTACATCATC
Reverse spi1b	TTACATGTAATGCTTTCTGTCTGTG
Forward *MLL*	GGACTACAAGGACGACGATGAC
Reverse *MLL*	CGGTCAGAGCCACTTCTAGGTC
Forward *ef1a*	GTGCTGTGCTGATTGTTGCT
Reverse *ef1a*	TGTATGCGCTGACTTCCTTG
Forward *tp53*	TTAAGTGATGTGGTGCCTGCCT
Reverse *tp53*	AGCTTCTTTCCCTGTTTGGGCT
Forward *mcl1a*	AACAAGAGCTGGCATGGGTT
Reverse *mcl1a*	CTTCTAAGCGCCTCGGTGAG

Table S1. Primers used in this study.
